# Virtual Scribes and Physician Time Spent on Electronic Health Records

**DOI:** 10.1001/jamanetworkopen.2024.13140

**Published:** 2024-05-24

**Authors:** Lisa Rotenstein, Edward R. Melnick, Christine Iannaccone, Jianyi Zhang, Aqsa Mugal, Stuart R. Lipsitz, Michael J. Healey, Christopher Holland, Richard Snyder, Christine A. Sinsky, David Ting, David W. Bates

**Affiliations:** 1 Harvard Medical School, Boston, Massachusetts; 2 Brigham and Women’s Hospital, Boston, Massachusetts; 3 University of California at San Francisco; 4 Department of Emergency Medicine, Yale School of Medicine, New Haven, Connecticut; 5 Department of Biostatistics (Health Informatics), Yale School of Public Health, New Haven, Connecticut; 6 Mass General Brigham, Boston, Massachusetts; 7 American Medical Association, Chicago, Illinois; 8 Massachusetts General Hospital, Boston; 9 Harvard School of Public Health, Boston, Massachusetts

## Abstract

**Importance:**

Time on the electronic health record (EHR) is associated with burnout among physicians. Newer virtual scribe models, which enable support from either a real-time or asynchronous scribe, have the potential to reduce the burden of the EHR and EHR-related documentation.

**Objective:**

To characterize the association of use of virtual scribes with changes in physicians’ EHR time and note and order composition and to identify the physician, scribe, and scribe response factors associated with changes in EHR time upon virtual scribe use.

**Design, Setting, and Participants:**

Retrospective, pre-post quality improvement study of 144 physicians across specialties who had used a scribe for at least 3 months from January 2020 to September 2022, were affiliated with Brigham and Women’s Hospital and Massachusetts General Hospital, and cared for patients in the outpatient setting. Data were analyzed from November 2022 to January 2024.

**Exposure:**

Use of either a real-time or asynchronous virtual scribe.

**Main Outcomes:**

Total EHR time, time on notes, and pajama time (5:30 pm to 7:00 am on weekdays and nonscheduled weekends and holidays), all per appointment; proportion of the note written by the physician and team contribution to orders.

**Results:**

The main study sample included 144 unique physicians who had used a virtual scribe for at least 3 months in 152 unique scribe participation episodes (134 [88.2%] had used an asynchronous scribe service). Nearly two-thirds of the physicians (91 physicians [63.2%]) were female and more than half (86 physicians [59.7%]) were in primary care specialties. Use of a virtual scribe was associated with significant decreases in total EHR time per appointment (mean [SD] of 5.6 [16.4] minutes; *P* < .001) in the 3 months after vs the 3 months prior to scribe use. Scribe use was also associated with significant decreases in note time per appointment and pajama time per appointment (mean [SD] of 1.3 [3.3] minutes; *P* < .001 and 1.1 [4.0] minutes; *P* = .004). In a multivariable linear regression model, the following factors were associated with significant decreases in total EHR time per appointment with a scribe use at 3 months: practicing in a medical specialty (−7.8; 95% CI, −13.4 to −2.2 minutes), greater baseline EHR time per appointment (−0.3; 95% CI, −0.4 to −0.2 minutes per additional minute of baseline EHR time), and decrease in the percentage of the note contributed by the physician (−9.1; 95% CI, −17.3 to −0.8 minutes for every percentage point decrease).

**Conclusions and Relevance:**

In 2 academic medical centers, use of virtual scribes was associated with significant decreases in total EHR time, time spent on notes, and pajama time, all per appointment. Virtual scribes may be particularly effective among medical specialists and those physicians with greater baseline EHR time.

## Key Points

**Question:**

What is the association of virtual scribe use with changes in physicians’ electronic health record (EHR) use patterns, and which factors are associated with changes in physicians’ EHR time upon use of a virtual scribe?

**Findings:**

In this quality improvement study of 144 physicians at 2 hospital systems, use of virtual scribes was associated with significant decreases in total EHR time, time on notes, and pajama time (5:30 pm to 7:00 am on weekdays and nonscheduled weekends and holidays), all per appointment. Practicing in a medical specialty, greater baseline EHR time, and greater decreases in note contribution by the physician were associated with significant decreases in total EHR time upon scribe use.

**Meaning:**

These findings suggest use of virtual scribes may be beneficial for influencing physicians’ EHR time, particularly among certain physicians.

## Teaser

This quality improvement study evaluates the association of use of virtual scribes with changes in physicians’ time spent on electronic health records.

## Introduction

Physician burnout is prevalent across the US.^1^ Organizational and system-level factors, including time spent interacting with electronic health records (EHRs), contribute to physicians’ burnout.^2^ Ambulatory physicians already spend more than half their days using the EHR and on EHR-based documentation, contributing to burnout.^3,4^ Scribes, who assist with clinical documentation and in some cases order entry, can alleviate burden associated with EHR-related tasks, thereby improving physicians’ experiences of providing care.^5,6,7^ While traditional scribe models involve having the scribe physically in the room with the physician and patient during a clinical visit, often with 1 physician working with only 1 scribe, this may be impractical at scale given cost constraints associated with in-person scribes and the logistical challenges of having another person physically present in the examination room.^8^ Newer virtual scribe models enable scribe support from either: (1) a synchronous (hereafter referred to as real time) virtual scribe who is present via a phone or teleconferencing technology during a patient visit, or (2) an asynchronous, virtual scribe who transcribes the documentation from a recording made during the visit.

Virtual documentation support has the potential to enhance the physician experience while reducing the barriers associated with in-person documentation assistance.^7,9^ Prior research in an ambulatory practice network demonstrated that remote, real-time scribes are associated with reductions in some EHR time measures, specifically among those physicians whose percentage of characters contributed to the note decreased with scribe use.^9^ In an academic primary care practice, physicians who used remote, real-time scribes had improvements in well-being metrics and total EHR time per 8 scheduled hours compared with controls who did not use virtual scribes.^7^ However, evidence from larger samples and for asynchronous scribes is limited. Additionally, it is not well understood which physician, EHR use pattern, or scribe factors are associated with reductions in EHR time and changes in the percentage of the note contributed by the physician, making it challenging to identify how to optimize virtual scribe usage.

In this study, we sought to answer 3 main research questions: (1) how is the use of virtual scribes associated with changes in EHR time and note and order composition by the physician; (2) what physician, scribe, and scribe response factors are associated with changes in EHR time upon virtual scribe use; and (3) what factors are associated with changes in the proportion of the note contributed by the physician upon virtual scribe use?

## Methods

### Study Setting

Since 2017, both Massachusetts General Hospital (MGH) and Brigham and Women’s Hospital (BWH) have experimented with asynchronous (DAX [Nuance], Scribble [IKS], Speke [Scribe America]) and real-time (tele-scribes) virtual scribe services. MGH and BWH serve as the main academic institutions comprising the Mass General Brigham integrated health care system in greater Boston, Massachusetts. Together, they deliver more than 3 million unique outpatient encounters annually. Availability of virtual scribe services for specific departments within MGH and BWH was determined by individual departmental leadership in partnership with information technology leadership. Physicians in the departments for which virtual scribes were offered could opt-in to trial scribe use, with trials in some instances funded by departments and in others requiring funding (or changes in clinical productivity) from the individual physician. This quality improvement study was approved by the Mass General Brigham institutional review board and followed Strengthening the Reporting of Observational Studies in Epidemiology (STROBE) reporting guidelines. All analyses were conducted with existing data sources, and thus, the institutional review board did not require participant to provide informed consent explicitly for this study.

### Study Participants

We first identified outpatient physicians at MGH and BWH who were currently using a virtual scribe as of September 2022 (and had been using a virtual scribe for at least 3 months) or who had used a virtual scribe between January 2020 and September 2022 for at least 3 months. The outpatient visits delivered by the physicians who are the focus of this study were a mix of in-person and telehealth visits. Since some physicians used more than one virtual scribe service (ie, some physicians used a real-time scribe, but then switched to an asynchronous scribe), we considered each virtual scribe use episode (defined as the time between starting and stopping a trial of a specific virtual scribe offering, based on administrative records regarding physicians’ trials of virtual scribe offerings) separately in analyses of changes in EHR time metrics and note and order contribution metrics. If a physician’s virtual scribe use episode lasted at least 3 months, the data for that episode were used in a pre-post 3-month analysis. If a physician’s virtual scribe use episode had lasted for 6 months or more, the data for that episode were included in a 6-month pre-post analysis as well. Of note, it was not possible to directly assess the percentage of visits during which a physician used the virtual scribe during each scribe use episode.

### Variables and Data Sources

For each physician, we extracted data about EHR use time (total time per appointment, time on notes per appointment, and pajama time per appointment [5:30 pm to 7:00 am on weekdays and nonscheduled weekends and holidays], number of appointments, proportion of the note written by the physician, and proportion of orders with team contributions) from our local Epic (Epic Systems) Signal database. Additionally, we noted whether physicians used an asynchronous vs synchronous or real-time virtual scribe service. We obtained information about each physician’s hospital affiliation and specialty from operational partners at each institution, and we obtained information regarding each physician’s sex and years since residency based on publicly available information in each institution’s physician directory or the physician’s Doximity listing.

### Statistical Analysis

We first used an interrupted time series method to assess whether our main outcome measure of total EHR time per appointment differed significantly month-to-month in the 3- and 6-month preintervention periods. This approach, meant to ensure that postintervention changes were not continuations of trends already seen in the preintervention period and thus unlikely attributable to the intervention, can be represented by the equation displayed in eFigure 1 in Supplement 1. We then used Wilcoxon signed rank tests to conduct physician-level comparisons of changes in total EHR time per appointment, note time per appointment, and pajama time per appointment, as well as the proportion of the note written by the physician and the proportion of orders with a team contribution at 3 months and 6 months before and after use of virtual scribes. In addition to conducting these analyses for the overall population of virtual scribe users, we conducted stratified analyses by scribe service type (real time vs asynchronous) and by specialty (primary care vs medical vs surgical specialty, as previously categorized^10^). To minimize the risk of type I error in the face of multiple comparisons, all *P* values for unadjusted analyses were corrected using the Benjamini-Hochberg method.^11^

All data were reported as frequencies (percentages) or means (SDs). While data on the proportion of the note written by the physician and the proportion of orders with a team contribution were available for all time points, Epic Signal data regarding the total number of appointments completed by a physician during a month were not available prior to August 2020, resulting in smaller sample sizes for pre-post analyses of EHR time metrics per appointment (numbers for each measure are shown in eTable 3 in Supplement 1).

To assess factors associated with physician-level 3-month changes in EHR use and time metrics, we built multivariable linear regression models with outcomes of (1) change in total EHR time per appointment, (2) change in notes time per appointment, and (3) change in pajama time per appointment after scribe use. Factors included in the model were baseline EHR time per appointment in the relevant time category for the model (total EHR time per appointment, notes time per appointment, or pajama time per appointment), change in the percentage of notes written by the physician, specialty, scribe service type (asynchronous vs real-time scribe), institution (BWH vs MGH), physician sex, the physician’s years in practice since residency (0 to 10 years, 11 to 20 years, or >20 years), and the change in the percentage of orders with team contribution after virtual scribe use. Finally, we built a multivariable model with an outcome of change in the percentage of the note contributed by the physician with scribe use. Factors included in this model were the baseline percentage of notes contributed by the physician, baseline note time per appointment, specialty, scribe service type, institution, physician sex, and years in practice since residency. All multivariable analyses were repeated for 6-month changes in total EHR time, notes time, pajama time, and changes in the proportion of the note contributed by the physician. Additionally, all models were repeated using quantile regression to assess for the robustness of model specifications to outliers.

An α of .05 was defined as statistical significance. All analyses were conducted in SAS version 9.4 (SAS Institute) and graphics were made in StataMP version 18 (StataCorp). Data were analyzed from November 2022 to January 2024.

## Results

### Sample Characteristics

A total of 144 unique physicians had used a virtual scribe for at least 3 months, representing 152 unique scribe participation episodes (Table 1). Nearly two-thirds of the physicians (91 physicians [63.2%]) were female. More than half of these physicians (86 physicians [59.7%]) were in primary care specialties, while about a third (46 physicians [32.0%]) were in medical specialties, with the balance (12 physicians [8.3%]) in surgical specialties. Most physicians had used an asynchronous scribe service (134 physicians [88.2%]) and practiced at MGH (123 physicians [85.4%]). About a third of physicians (43 physicians [35.5%]) in the sample were 0 to 10 years from completion of residency, 36 (29.8%) were 11 to 20 years from residency, and 42 (34.7%) were more than 20 years from the completion of residency. Medical specialists (12 physicians [23.5%]) and surgical specialists (3 physicians [23.1%]) were more likely to use a synchronous scribe service than primary care physicians (3 physicians [3.4%]). Users of asynchronous as compared with synchronous scribes did not differ significantly in terms of practice site, sex, or years since residency. Characteristics of the 121 physicians who had 122 unique scribe participation episodes for 6 or more months are shown in eTable 1 in Supplement 1.

**Table 1.** Characteristics of Physicians in Electronic Health Record Metrics 3-Month Analytic Sample

Characteristic	Physicians, No. (%) (N = 144)
Sex	
Male	53 (36.8)
Female	91 (63.2)
Specialty category	
Medical specialty	46 (32.0)
Primary care	86 (59.7)
Surgical specialty	12 (8.3)
Hospital	
Brigham and Women’s Hospital	21 (14.6)
Massachusetts General Hospital	123 (85.4)
Years since residency	
<5	11 (7.6)
5-10	38 (26.4)
11-15	26 (18.1)
16-20	21 (14.6)
21-25	18 (12.5)
26-29	10 (6.9)
≥30	20 (13.9)
Scribe service^a^	
Asynchronous	134 (88.2)
Real-time	18 (11.8)

^a^ Out of 152 unique scribe participation episodes.

### Changes in EHR Time, Note Contribution, and Order Contribution Measures in Overall Cohort

**Figure 1.**
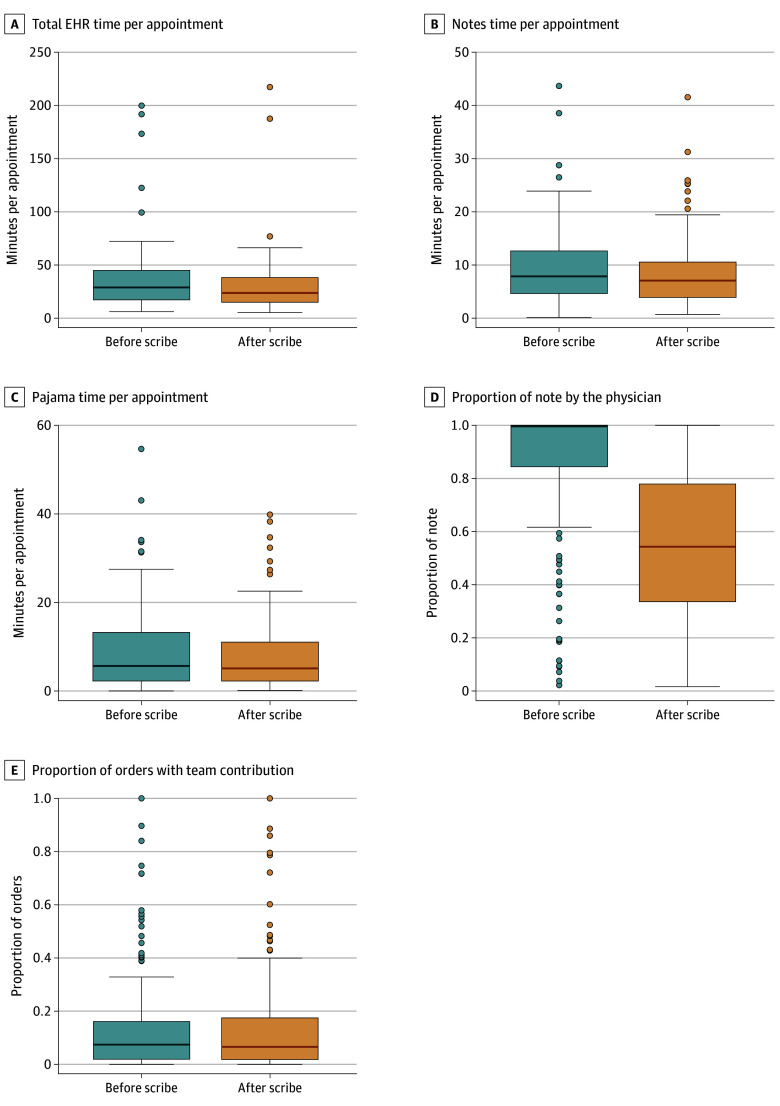
Three-Month Change in Electronic Health Record (EHR) Metrics With Scribe Use for Overall Cohort The lower edge of the box represents quartile 1, the upper edge of the box represents quartile 3, and the middle line through the box represents the median. Dots represent outliers.

There was no significant month-to-month change in total EHR time per appointment in the 3 months (β = 0.005; 95% CI, −0.018 to 0.009; *P* = .48) or 6 months (β = 0.006; 95% CI, −0.003 to 0.016; *P* = .18) before virtual scribe adoption. As shown in Figure 1 and eTable 2 in Supplement 1, for the overall sample, virtual scribe use was associated with significant decreases in total EHR time per appointment (mean [SD] of 5.6 [16.4] minutes; *P* < .001) in the 3 months after vs the 3 months before scribe use. Virtual scribe use was also associated with significant decreases in note time per appointment and pajama time per appointment (mean [SD] change of 1.3 [3.3] minutes; *P* < .001 and 1.1 [4.0] minutes; *P* = .004, respectively). Across the sample, virtual scribe use was associated with a significant change in the proportion of the note contributed by the physician, which decreased by a mean (SD) of 0.3 (0.3) minutes (*P* < .001), but not in the proportion of orders with team contribution (mean [SD] difference of 0.0 [0.1] minutes; *P* = .72). Similar trends were observed in 6-month pre-post analyses (eFigure 3 in Supplement 1).

### Changes in EHR Time, Note Contribution, and Order Contribution Measures by Specialty

**Figure 2.**
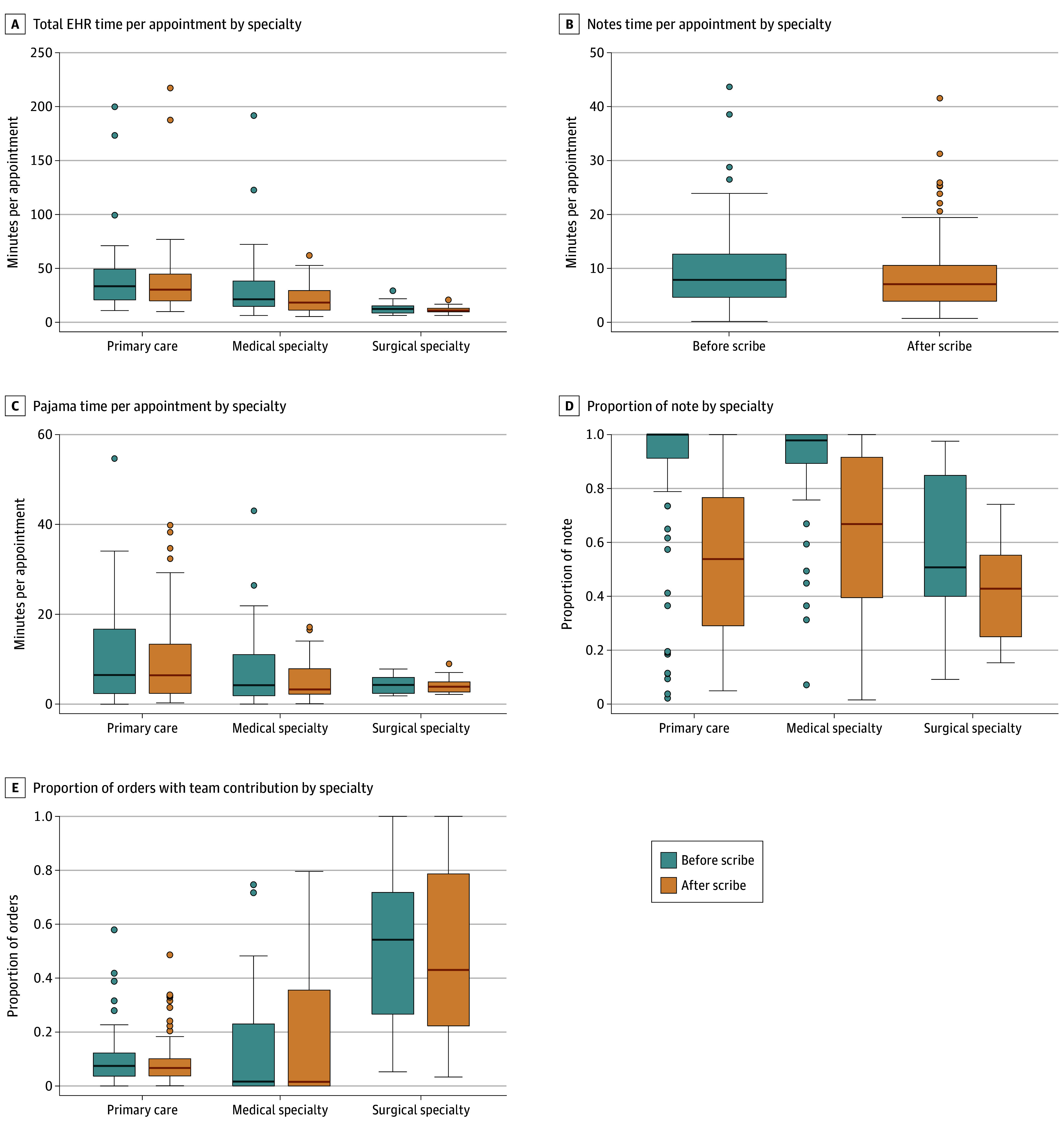
Three-Month Change in Electronic Health Record (EHR) Metrics With Scribe Use by Specialty The lower edge of the box represents quartile 1, the upper edge of the box represents quartile 3, and the middle line through the box represents the median. Dots represent outliers.

As shown in Figure 2 and eTable 2 in Supplement 1, in unadjusted analyses stratified by specialty, use of virtual scribes was associated with significant 3-month decreases in total EHR time per appointment among primary care and medical specialists but not surgical specialists (primary care: mean [SD] difference of −3.3 [10.2] minutes; *P* < .001; medical specialists: mean [SD] −11.0 [24.8] minutes; *P* < .001; surgical specialists: mean [SD] −1.7 [4.6] minutes; *P* = .65). Similarly, use of virtual scribes was associated with significant 3-month decreases in note time per appointment among primary care and medical specialists but not surgical specialists (primary care: mean [SD] difference of −1.5 [3.5] minutes; *P* < .001; medical specialists: mean [SD] −1.4 [3.0] minutes; *P* = .001; surgical specialists: mean [SD] 0.4 [1.5] minutes; *P* = .65). Use of virtual scribes was associated with significant 3-month decreases in pajama time per appointment for medical specialists (mean [SD] difference of −2.2 [5.3] minutes; *P* = .01), but not for primary care specialists (mean [SD] −0.7 [3.5] minutes; *P* = .18) or for surgical specialists (mean [SD] −0.2 [1.4] minutes; *P* = .68). Changes in the proportion of the note contributed by the physician and the proportion of orders with team contribution are shown in eTable 2 in Supplement 1. Similar trends were seen in 6-month pre-post analyses stratified by specialty (eFigure 4 in Supplement 1).

### Changes in EHR Time, Note Contribution, and Order Contribution Measures by Scribe Service Type

In unadjusted analyses stratified by scribe service type (eFigure 2 and eTable 2 in Supplement 1), virtual scribe use was associated with significant 3-month decreases in total EHR time per appointment with asynchronous but not with real-time scribes (asynchronous scribes: mean [SD] difference of −3.9 [12.3] minutes; *P* = .002; real-time scribes: mean [SD] difference of −18.4 [32.3] minutes; *P* = .003). While use of both real-time and asynchronous scribes was associated with numerical decreases in note time per appointment, the change only reached significance for asynchronous scribes (asynchronous scribes: mean [SD] difference of −1.3 [3.4] minutes; *P* < .001; real-time scribes: mean [SD] difference of −1.2 [2.2] minutes; *P* = .09). Use of both asynchronous and real-time virtual scribes was associated with significant changes in pajama time per appointment (asynchronous scribes: mean [SD] change of −0.9 [4.1] minutes; *P* = .05; real-time scribes: mean [SD] of −2.9 [3.7] minutes; *P* = .002), as well as changes in the proportion of the note contributed by the physician (asynchronous scribes: mean [SD] difference of −0.3 [0.3]; *P* < .002; real-times scribes: mean [SD] −0.4 [0.2]; *P* = .03). Changes in the proportion of the note contributed by the physician and the proportion of orders with team contribution are shown in eTable 2 in Supplement 1. Similar trends were seen in 6-month pre-post analyses stratified by scribe type (eFigure 5 in Supplement 1).

### Factors Associated With Changes in EHR Time Measures and Note Contribution by the Physician

As shown in Table 2, in a multivariable linear regression model, the following factors were associated with significant decreases in the outcome of change in total EHR time per appointment with scribe use at 3 months: practicing in a medical specialty (−7.8; 95% CI, −13.4 to −2.2 minutes; *P* = .01), greater baseline EHR time per appointment (−0.3; 95% CI, −0.4 to −0.2 minutes per additional minute of baseline EHR time; *P* < .001), and reductions in the percentage of the note contributed by the physician (9.1; 95% CI, 17.3 to 0.8 minutes for every percentage point reduction; *P* = .03). In a multivariable model with an outcome of total notes time per appointment (Table 2), the following factors were associated with greater decreases after scribe use: greater baseline time on notes per appointment (−0.2; 95% CI, −0.3 to −0.1 minutes per additional minute of baseline note time; *P* < .001) and reductions in the percentage of the note contributed by the physician (−5.8; 95% CI, −7.4 to −4.2 minutes for every percentage point reduction; *P* < .001). Practicing in a medical specialty (−2.2; 95% CI, −3.6 to −0.8 minutes; *P* = .002) and greater baseline pajama time per appointment was associated with a greater decrease in the total pajama time per appointment with scribe use (−0.2; 95% CI, −0.3 to −0.2 minutes per additional minute of baseline pajama time; *P* < .001) (Table 2). Corresponding results for 6-month changes in EHR time variables are shown in eTable 3 in Supplement 1.

**Table 2.** Factors Associated in Multivariable Linear Regression Model With 3-Month Change in Total Electronic Health Record (EHR) Time, Notes Time, and Pajama Time, All per Appointment, After Scribe Use

Parameter	Total EHR time per appointment		Notes time per appointment		Pajama time per appointment	
	Estimate (95% CI), min per visit	*P* value	Estimate (95% CI), min per visit	*P* value	Estimate (95% CI), min per visit	*P* value
Baseline EHR time per appointment, min	−0.29 (−0.37 to −0.21)	<.001	−0.18 (−0.25 to −0.11)	<.001	−0.25 (−0.31 to −0.18)	<.001
Change in % of note contribution by the physician	−9.07 (−17.33 to −0.81)	.03	5.83 (4.23 to 7.42)	<.001	0.78 (−1.31 to 2.86)	.47
Specialty						
Medical specialty	−7.79 (−13.35 to −2.24)	.01	−0.45 (−1.53 to 0.63)	.41	−2.17 (−3.56 to −0.78)	.002
Surgical specialty	−6.34 (−15.53 to 2.86)	.18	−0.35 (−2.09 to 1.38)	.69	−0.95 (−3.15 to 1.26)	.40
Primary care	[Reference]	NA	[Reference]	NA	[Reference]	NA
Scribe service type						
Asynchronous	6.80 (−1.44 to 15.04)	.11	−1.05 (−2.62 to 0.52)	.19	0.58 (−1.42 to 2.59)	.57
Real-time scribe	[Reference]	NA	[Reference]	NA	[Reference]	NA
Institution						
Brigham and Women’s Hospital	−0.09 (−6.96 to 6.78)	.98	−0.51 (−1.83 to 0.80)	.45	0.96 (−0.78 to 2.69)	.28
Massachusetts General Hospital	[Reference]	NA	[Reference]	NA	[Reference]	NA
Physician sex						
Female	−0.54 (−5.54 to 4.45)	.83	0.31 (−0.67 to 1.28)	.54	−0.09 (−1.34 to 1.16)	.89
Male	[Reference]	NA	[Reference]	NA	[Reference]	NA
Years since residency						
0-10	0.98 (−4.83 to 6.80)	.73	0.18 (−0.89 to 1.25)	.74	0.25 (−0.91 to 2.02)	.46
11-20	−2.44 (−8.33 to 3.44)	.42	−0.38 (−1.52 to 0.75)	.51	0.55 (−1.11 to 1.62)	.72
>20	[Reference]	NA	[Reference]	NA	[Reference]	NA
Change in the percentage of orders with team contribution	−4.09 (−36.89 to 28.70)	.81	−1.17 (−7.44 to 5.10)	.71	−2.20 (−10.21 to 5.81)	.59

Abbreviation: NA, not applicable.

Finally, in a multivariable linear regression model with an outcome of change in the proportion of the note contributed by the physician after scribe use (Table 3), only a greater baseline percentage of the note by the physician was associated with a greater decrease in this outcome (−0.6%; 95% CI, −0.7% to −0.4% for every percentage of the note written by the physician at baseline; *P* < .001). Results for 6-month changes in proportion of the note contributed by the physician are shown in eTable 4 in Supplement 1. For all model specifications, consistent results were seen in models limited to asynchronous scribe users (results not shown) and directionally similar models were obtained using quantile regression (results not shown).

**Table 3.** Factors Associated in Multivariable Linear Regression Model With 3-Month Change in Proportion of the Note by the Physician

Parameter	Estimate (95% CI), min per visit	*P *value
Baseline percentage of note by the physician	−0.56 (−0.73 to −0.38)	<.001
Baseline note time per appointment	0.01 (0.00 to 0.01)	.07
Specialty		
Medical specialty	0.05 (−0.05 to 0.16)	.31
Surgical specialty	0.07 (−0.10 to 0.24)	.42
Primary care	[Reference]	NA
Scribe service type		
Asynchronous	0.11 (−0.03 to 0.26)	.12
Real-time scribe	[Reference]	NA
Institution		
Brigham and Women’s Hospital	−0.02 (−0.14 to 0.11)	.80
Massachusetts General Hospital	[Reference]	NA
Physician sex		
Female	0.01 (−0.09 to 0.11)	.88
Male	[Reference]	NA
Years since residency		
0-10	−0.06 (−0.16 to 0.04)	.25
11-20	0.02 (−0.09 to 0.13)	.73
>20	[Reference]	NA

Abbreviation: NA, not applicable.

## Discussion

In this cohort study across 2 academic medical centers, we found that use of virtual scribes is associated with significant decreases in total EHR time per appointment, time on notes per appointment, and pajama time per appointment at both 3 and 6 months of scribe use. These associations were seen with use of both real-time and asynchronous scribes and among primary care and medical specialists. Factors significantly associated with decreases in EHR time per appointment upon scribe use included greater baseline EHR time, a greater decrease in the proportion of the note contributed by the physician, and practicing in a medical specialty. Physicians with a greater baseline contribution to their notes had larger decreases in the proportion of the note written by the physician after scribe use. Notably, virtual scribe use was not associated with significant changes in the proportion of orders with a team contribution.

Multiple studies have demonstrated the association of use of in-person scribes in the ambulatory setting with less time spent on the EHR,^6,12^ as well as with improved physician satisfaction with the EHR,^12^ increased clinical productivity,^13^ and enhanced physician satisfaction with their patient interactions.^5,6^ However, in-person scribes have many limitations, including labor costs, labor supply, and having someone else present in the examination room.^8^ Virtual scribe services have the potential to bypass these constraints. Our results demonstrate the ability of virtual scribes to reduce physicians’ time on the EHR, and thus to help reduce the burden of the EHR for physicians. These results are consistent with prior studies in single practices or with single vendors demonstrating positive associations between virtual scribes and EHR time expenditure.

However, despite these benefits of virtual scribes, there are still up-front and ongoing costs related to their implementation,^14^ and prior evidence suggests that only a proportion of physicians respond to virtual scribes as defined by the proportion of the note contributed by the physician decreasing and EHR time decreasing accordingly.^9^ Thus, it is valuable to identify which factors are associated with greater benefit from virtual scribes, at least from a numeric perspective. In this study, practicing in a medical specialty, having higher baseline EHR time per appointment, and experiencing greater reductions in the percentage of the note contributed by the physician upon scribe use were all associated with greater reductions in EHR time upon scribe use. A higher percentage of the note contributed by the physician at baseline was the main factor significantly associated with reductions in note contribution by the physician. Together, these findings suggest that physicians with a greater EHR time burden at baseline and who receive minimal documentation support from other team members (eg, medical assistants or physician assistants) may stand to benefit most from virtual scribe technology. Organizations implementing scribes may want to consider prioritizing these physicians in their rollout processes. Notably, our stratified analyses demonstrated lower baseline EHR time per appointment and lower baseline physician note contributions by surgical specialists. These findings suggest that the physicians who most stand to benefit from virtual scribe technology may not be those whose specialties most directly experience the productivity and financial benefits of scribes.^15,16^

Our findings also highlight potential limits to the benefits of virtual scribes, particularly those without a synchronous component. To our surprise, virtual scribes were not associated with significant changes in the proportion of orders with a team contribution in the present analysis. Although there was a trend toward an increase in this measure among real-time scribe users, this difference did not reach statistical significance. In-person medical scribes often perform nondocumentation actions such as pending orders, which may further reduce physicians’ EHR burden.^17,18^ More recently, in a study by Micek et al,^7^ use of audio-only scribes who documented in real-time was associated with an average 10% increase in the percentage of orders with a team contribution. We have previously found that above-average team contribution to orders is associated with lower EHR time across multiple categories for physicians.^19^ While asynchronous virtual scribe solutions, in which an encounter is recorded and subsequently transcribed either by a remote individual or via AI–based technology, obviate the need for an individual to be present in the examination room or to coordinate a remote individual being virtually present for a particular visit, a fully asynchronous model may limit the ability of scribes to aid physicians with nondocumentation functions that further speed workflows. These limitations likely compound support lost in the transition from in-person to virtual scribes, such as the ability to help physicians find relevant information in the EHR, suggest diagnoses for coding, or help physicians meet patients’ nonclinical needs (eg, wayfinding) during the visit.

In the future, it is likely that the capacity of virtual scribes will be greatly enhanced using predominantly AI-powered solutions. It is possible that these tools’ best performance will be achieved by using AI to develop a note that is then reviewed by a remote human scribe and only subsequently reviewed by a physician. While further work is needed to ensure the accuracy of these tools’ output and their relative benefits,^20^ when optimized, these tools have the capacity to extend the identified benefits of virtual scribing to many more medical encounters.

### Limitations and Strengths

This study has multiple limitations. First, it is based on virtual scribe implementation in 2 academic medical centers. The documentation and EHR time expenditure patterns of physicians in these centers may not represent those of nonacademic physicians. Our study population included mostly asynchronous scribe users and physicians practicing at MGH, limiting our ability to draw well-powered conclusions about specific subgroups, such as those using real-time scribes or those practicing at BWH. Additionally, the data for this study were collected prior to the use of AI without human review for scribing, and thus results for purely AI-powered solutions may differ. The analysis is limited by its pre-post design. This design choice was influenced by the fact that only certain physicians at BWH and MGH chose to use virtual scribes. These physicians were likely different in distinct ways from those who did not adopt scribes, and thus would not have been a comparable control group. This additionally suggests that if virtual scribes were provided to all physicians in an organization, including those who may not have voluntarily opted into a scribe use program, results may differ from ours. Finally, much of the study took place during the most active periods of the COVID-19 pandemic. This time period likely saw multiple shifts in physicians’ EHR time given an increase in telehealth use and concomitant decrease in in-person visits, as well as an increase in inbox message volume.^21^ These limitations are balanced by several strengths. These include a longitudinal study design, the use of multiple virtual scribing technologies among the study population, the representation of multiple specialty types, and the assessment of our study outcomes at both 3-month and 6-month time points. Additionally, we were able to obtain granular information about physicians’ EHR use patterns over time, as well as the relative contribution of these physicians to notes and orders at multiple time points. Future studies should delve deeper into the subjective experiences of these physicians to understand how numeric benefits of virtual scribes compare with perceived benefits among physician adopters.

## Conclusions

In 2 academic medical centers, we found that use of virtual scribes was associated with significant decreases in total EHR time per appointment, time spent on notes per appointment, and pajama time per appointment. Virtual scribes may be particularly effective among medical specialists and those physicians with greater baseline EHR time. Our findings demonstrate the benefits of virtual scribes and identify the physicians for whom they may be most effective. Further work is needed in this rapidly evolving area if we are to reduce the rates of burnout at scale.

## Notes

Supplement 1. **eFigure 1. **Equation for Interrupted Time Series Methodology **eTable 1. **Characteristics of Physicians in EHR Metrics 6-Month Analytic Sample **eTable 2. **EHR Use and Time Expenditure Patterns for 3 Months Pre- vs Postscribe Use **eFigure 2. **3-Month Change in EHR Metrics With Scribe Use, Scribe Service Type **eFigure 3. **6-Month Change in EHR Metrics With Scribe Use for Overall Cohort **eFigure 4. **6-Month Change in EHR Metrics With Scribe Use, Stratified by Specialty **eFigure 5. **6-Month Change in EHR Metrics With Scribe Use, Stratified by Scribe Service Type **eTable 3. **Factors Associated With 6-Month Change **eTable 4. **Factors Associated With 6-Month Change in Proportion of the Note by the Physician

Supplement 2.
Data Sharing Statement

## References

[1] National Academies of Sciences, Engineering, and Medicine. taking action against clinician burnout:a systems approach to professional well-being. Accessed April 17, 2024. https://nap.nationalacademies.org/catalog/25521/taking-action-against-clinician-burnout-a-systems-approach-to-professional 31940160

[2] DeChant PF, Acs A, Rhee KB, . Effect of organization-directed workplace interventions on physician burnout: a systematic review. Mayo Clin Proc Innov Qual Outcomes. 2019;3(4):384-408. doi:10.1016/j.mayocpiqo.2019.07.006 31993558 PMC6978590

[3] Arndt BG, Beasley JW, Watkinson MD, . Tethered to the EHR: primary care physician workload assessment using EHR event log data and time-motion observations. Ann Fam Med. 2017;15(5):419-426. doi:10.1370/afm.2121 28893811 PMC5593724

[4] Sinsky C, Colligan L, Li L, . Allocation of physician time in ambulatory practice: a time and motion study in 4 specialties. Ann Intern Med. 2016;165(11):753-760. doi:10.7326/M16-0961 27595430

[5] Gidwani R, Nguyen C, Kofoed A, . Impact of scribes on physician satisfaction, patient satisfaction, and charting efficiency: a randomized controlled trial. Ann Fam Med. 2017;15(5):427-433. doi:10.1370/afm.2122 28893812 PMC5593725

[6] Mishra P, Kiang JC, Grant RW. Association of medical scribes in primary care with physician workflow and patient experience. JAMA Intern Med. 2018;178(11):1467-1472. doi:10.1001/jamainternmed.2018.3956 30242380 PMC6248201

[7] Micek MA, Arndt B, Baltus JJ, . The effect of remote scribes on primary care physicians’ wellness, EHR satisfaction, and EHR use. Healthc (Amst). 2022;10(4):100663. doi:10.1016/j.hjdsi.2022.100663 36375356

[8] Bates DW, Landman AB. Use of medical scribes to reduce documentation burden: are they where we need to go with clinical documentation? JAMA Intern Med. 2018;178(11):1472-1473. doi:10.1001/jamainternmed.2018.3945 30242315

[9] Ong SY, Moore JM, Williams B, O’Connell RT, Goldstein R, Melnick ER. How a virtual scribe program improves physicians’ EHR experience, documentation time, and note quality. NEJM Catal. 2021;2(12). doi:10.1056/CAT.21.0294

[10] Rotenstein LS, Holmgren AJ, Downing NL, Bates DW. Differences in total and after-hours electronic health record time across ambulatory specialties. JAMA Intern Med. 2021;181(6):863-865. doi:10.1001/jamainternmed.2021.0256 33749732 PMC7985815

[11] Haynes W. Benjamini–Hochberg Method. In: Dubitzky W, Wolkenhauer O, Cho KH, Yokota H, eds. Encyclopedia of Systems Biology. Springer; 2013:78-78.

[12] Pozdnyakova A, Laiteerapong N, Volerman A, . Impact of medical scribes on physician and patient satisfaction in primary care. J Gen Intern Med. 2018;33(7):1109-1115. doi:10.1007/s11606-018-4434-6 29700790 PMC6025675

[13] Zallman L, Finnegan K, Roll D, Todaro M, Oneiz R, Sayah A. Impact of medical scribes in primary care on productivity, face-to-face time, and patient comfort. J Am Board Fam Med. 2018;31(4):612-619. doi:10.3122/jabfm.2018.04.170325 29986987

[14] Ghatnekar S, Faletsky A, Nambudiri VE. Digital scribe utility and barriers to implementation in clinical practice: a scoping review. Health Technol (Berl). 2021;11(4):803-809. doi:10.1007/s12553-021-00568-0 34094806 PMC8169416

[15] McCormick BJ, Deal A, Borawski KM, . Implementation of medical scribes in an academic urology practice: an analysis of productivity, revenue, and satisfaction. World J Urol. 2018;36(10):1691-1697. doi:10.1007/s00345-018-2293-8 29637266

[16] Miksanek TJ, Skandari MR, Ham SA, . The productivity requirements of implementing a medical scribe program. Ann Intern Med. 2021;174(1):1-7. doi:10.7326/M20-0428 33017564

[17] Rule A, Chiang MF, Hribar MR. Medical scribes have a variable impact on documentation workflows. IOS Press. Accessed April 17, 2024. https://ebooks.iospress.nl/doi/10.3233/SHTI220208 10.3233/SHTI220208 PMC10477084 35673147

[18] Nambudiri VE, Watson AJ, Buzney EA, Kupper TS, Rubenstein MH, Yang FC. Medical scribes in an academic dermatology practice. JAMA Dermatol. 2018;154(1):101-103. doi:10.1001/jamadermatol.2017.3658 29094159 PMC5833578

[19] Rotenstein LS, Holmgren AJ, Horn DM, . System-level factors and time spent on electronic health records by primary care physicians. JAMA Netw Open. 2023;6(11):e2344713. doi:10.1001/jamanetworkopen.2023.44713 37991757 PMC10665969

[20] van Buchem MM, Boosman H, Bauer MP, Kant IMJ, Cammel SA, Steyerberg EW. The digital scribe in clinical practice: a scoping review and research agenda. NPJ Digit Med. 2021;4(1):57. doi:10.1038/s41746-021-00432-5 33772070 PMC7997964

[21] Holmgren AJ, Downing NL, Tang M, Sharp C, Longhurst C, Huckman RS. Assessing the impact of the COVID-19 pandemic on clinician ambulatory electronic health record use. J Am Med Inform Assoc. 2022;29(3):453-460. doi:10.1093/jamia/ocab268 34888680 PMC8689796

