# Supplementary material for: Virtual Scribes and Physician Time Spent on Electronic Health Records
Source: JAMA Netw Open. 2024 May 24;7(5):e2413140. doi: 10.1001/jamanetworkopen.2024.13140 (PMC11127114; doi:10.1001/jamanetworkopen.2024.13140)
Supplement: Supplement 1. — eFigure 1. Equation for Interrupted Time Series Methodology eTable 1. Characteristics of Physicians in EHR Metrics 6-Month Analytic Sample eTable 2. EHR Use and Time Expenditure Patterns for 3 Months Pre- vs Postscribe Use eFigure 2. 3-Month Change in EHR Metrics With Scribe Use, Scribe Service Type eFigure 3. 6-Month Change in EHR Metrics With Scribe Use for Overall Cohort eFigure 4. 6-Month Change in EHR Metrics With Scribe Use, Stratified by Specialty eFigure 5. 6-Month Change in EHR Metrics With Scribe Use, Stratified by Scribe Service Type eTable 3. Factors Associated With 6-Month Change eTable 4. Factors Associated With 6-Month Change in Proportion of the Note by the Physician [file jamanetwopen-e2413140-s001.pdf]

Supplemental Online Content

Rotenstein L, Melnick ER, Iannaccone C, et al. Virtual scribes and physician time spent on electronic health records. *JAMA Netw Open*. 2024;7(5):e2413140.
doi:10.1001/jamanetworkopen.2024.13140

eFigure 1. Equation for Interrupted Time Series Methodology
eTable 1. Characteristics of Physicians in EHR Metrics 6-Month Analytic Sample
eTable 2. EHR Use and Time Expenditure Patterns for 3 Months Pre- vs. Postscribe Use
eFigure 2. 3-Month Change in EHR Metrics With Scribe Use, Scribe Service Type
eFigure 3. 6-Month Change in EHR Metrics with Scribe Use for Overall Cohort
eFigure 4. 6-Month Change in EHR Metrics with Scribe Use, Stratified by Specialty
eFigure 5. 6-Month Change in EHR Metrics with Scribe Use, Stratified by Scribe Service Type
eTable 3. Factors Associated with 6-Month Change
eTable 4. Factors Associated with 6-Month Change in Proportion of the Note by the Physician
eFigure 5. Pajama Time per Appointment by Scribe Type

This supplemental material has been provided by the authors to give readers additional information about their work.

© 2024 Rotenstein L et al. *JAMA Network Open.*

eFigure 1. Equation for Interrupted Time Series Methodology (segmented linear regression model for change in level and slope from pre to post period)

$Y_t = \beta_0 + \beta_1 t + \beta_2 D_t + \beta_3 (t\text{-}T1)D_t + \varepsilon_t.$

- Y_t is the outcome measured at time t, with the "interruption" or intervention occurring at time T_1
- D_t represents the post-intervention interval. It has a value of 0 in the pre-intervention period and a value of 1 in the post-intervention period.
- β_0 is the intercept
- β_1 is the pre-intervention slope
- β_2 is the change in the level from the pre-intervention to the post-intervention period
- β_3 is the change in the slope from the pre-interruption slope to the post-interruption slope

© 2024 Rotenstein L et al. *JAMA Network Open.*

eTable 1. Characteristics of Physicians in EHR Metrics 6-Month Analytic Sample

Characteristic	N	%
**Gender**<br>*(N=121 unique physicians)*		
Male	48	39.7
Female	73	60.3
**Specialty Category**<br>*(N=121 unique physicians)*		
Medical Specialty	36	29.8
Primary Care	75	62.0
Surgical Specialty	10	8.2
**Hospital**<br>*(N=121 unique physicians)*		
BWH	16	13.2
MGH	105	86.8
**Years Since Residency**<br>*(N=121 unique physicians)*		
< 5 years	9	7.4
5 -10 years	34	28.1
11-15 years	20	16.6
16-20 years	16	13.2
21-25 years	14	11.6
26-29 years	9	7.4
30+ years	19	15.7
**Scribe Service**<br>*(N=122 unique scribe participation episodes)*		
Asynchronous	108	88.5
Real-Time	14	11.5

© 2024 Rotenstein L et al. *JAMA Network Open.*

eTable 2. EHR Use and Time Expenditure Patterns for 3 Months Pre- vs Post-Scribe Use a) Overall Cohort, b) by Specialty, c) by Scribe Service Type

a) Overall Cohort

	3 Months Pre-Scribe Use	3 Months Post-Scribe Use	P-Value for Difference	Corrected P-Value	N
	Median (Q1, Q3)	Median (Q1, Q3)			
**Total EHR Time Per Appointment (minutes)**	29.0 (17.02, 45.0)	23.2 (14.9, 36.8)	<0.001	<0.001	129
**Note Time Per Appointment (minutes)**	7.9 (4.6, 12.6)	7.1 (3.9, 10.6)	< 0.001	<0.001	127
**Pajama Time Per Appointment (minutes)**	5.6 (2.3, 13.2)	5.1 (2.2, 11.0)	0.003	0.004	126
**Proportion of Note Contributed by Provider**	1.00 (0.84, 1.00)	0.54 (0.34, 0.78)	<0.001	<0.001	152
**Proportion of Orders with Team Contribution**	0.07 (0.02, 0.16)	0.07 (0.02, 0.17)	0.72	0.72	152

b) by Specialty

	3 Months Pre-Scribe Use	3 Months Post-Scribe Use	P-Value for Difference	Corrected [a] P-Value	N
	Median (Q1, Q3)	Median (Q1, Q3)			
Medical Specialty					
**Total EHR Time Per Appointment (minutes)**	21.2 (14.6, 38.1)	17.5 (11.2, 24.5)	<0.001	<0.001	41
**Note Time Per Appointment (minutes)**	8.20 (5.90, 12.40)	7.10 (3.70, 10.30)	0.0006	0.001	40
**Pajama Time Per Appointment (minutes)**	4.20 (1.90, 11.00)	3.60 (2.00, 8.00)	0.005	0.006	40
**Proportion of Note Contributed by Provider**	0.98 (0.89, 1.0)	0.67 (0.40, 0.92)	<0.001	<0.001	51
**Proportion of Orders with Team Contribution**	0.02 (0.00, 0.23)	0.02 (0.00, 0.36)	0.20	0.20	51
Surgical Specialty					
**Total EHR Time Per Appointment (minutes)**	12.3 (8.5, 15.1)	10.8 (9.6, 13.0)	0.38	0.65	76
**Note Time Per Appointment (minutes)**	4.50 (2.20, 7.60)	4.40 (2.30, 7.50)	0.42	0.65	75
**Pajama Time Per Appointment (minutes)**	4.30 (2.40, 6.00)	4.00 (2.70, 5.00)	0.68	0.68	74
**Proportion of Note Contributed by Provider**	0.51 (0.40, 0.85)	0.43 (0.25, 0.55)	0.05	0.25	88
**Proportion of Orders with Team Contribution**	0.54 (0.27, 0.72)	0.43 (0.22, 0.79)	0.52	0.65	88
Primary Care Specialty					
**Total EHR Time Per Appointment (minutes)**	33.3 (20.6, 49.3)	30.2 (19.8, 45.9)	0.0003	0.0005	12
**Note Time Per Appointment (minutes)**	8.60 (5.70, 14.00)	8.00 (4.60, 12.00)	0.0002	0.0005	12
**Pajama Time Per Appointment (minutes)**	6.50 (2.40, 16.70)	6.40 (2.40, 13.70)	0.14	0.18	12
**Proportion of Note Contributed by Provider**	1.00 (0.91, 1.00)	0.54 (0.29, 0.77)	<0.001	0.0005	13
**Proportion of Orders with Team Contribution**	0.07 (0.04, 0.12)	0.07 (0.04, 0.1)	0.34	0.34	13

© 2024 Rotenstein L et al. *JAMA Network Open.*

c) by Scribe Service Type

	3 Months Pre-Scribe Use	3 Months Post-Scribe Use	P-Value for Difference	Corrected [a] P-Value	N
	Median (Q1, Q3)	Median (Q1, Q3)			
Asynchronous					
**Total EHR Time Per Appointment (minutes)**	28.5 (17.2, 45.0)	24.4 (15.7, 37.1)	<0.001	0.002	15
**Note Time Per Appointment (minutes)**	7.8 (4.6, 12.6)	7.1 (3.9, 10.5)	< 0.001	0.002	15
**Pajama Time Per Appointment (minutes)**	5.5 (2.0, 13.2)	5.0 (2.2, 11.4)	0.04	0.05	15
**Proportion of Note Contributed by Provider**	1.00 (0.85, 1.00)	0.54 (0.36, 0.79)	< 0.001	0.002	18
**Proportion of Orders with Team Contribution**	0.07 (0.02, 0.16)	0.06 (0.02, 0.13)	0.31	0.31	18
Real-Time					
**Total EHR Time Per Appointment (minutes)**	29.2 (24.4, 54.1)	18.9 (10.7, 32.0)	0.0006	0.003	114
**Note Time Per Appointment (minutes)**	8.3 (4.7, 15.3)	9.5 (2.3, 13.5)	0.07	0.09	112
**Pajama Time Per Appointment (minutes)**	6.6 (2.5, 14.1)	5.5 (2.2, 8.8)	0.004	0.02	111
**Proportion of Note Contributed by Provider**	0.97 (0.76, 1.0)	0.58 (0.32, 0.67)	< 0.001	0.03	134
**Proportion of Orders with Team Contribution**	0.17 (0.22)	0.22 (0.24)	0.13	0.13	134

a. P-values corrected for multiple comparisons using the Benjamini-Hochberg method.

© 2024 Rotenstein L et al. *JAMA Network Open.*

eFigure 2. Three-Month Change in EHR Metrics With Scribe Use, Scribe Service Type

Total EHR Time Per Appointment

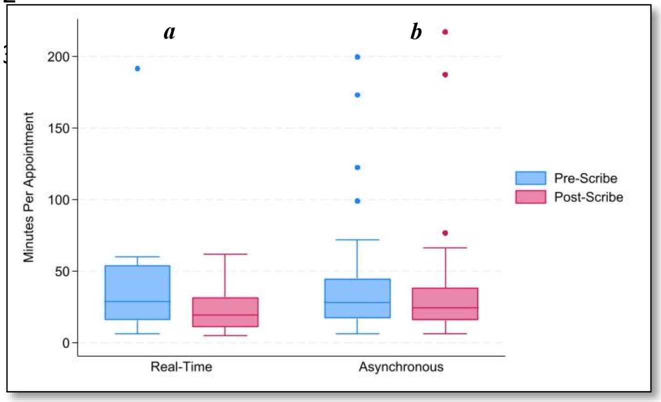

Note Time Per Appointment

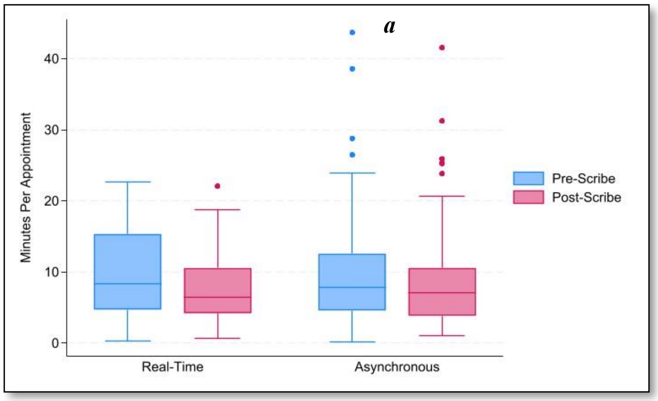

Pajama Time Per Appointment

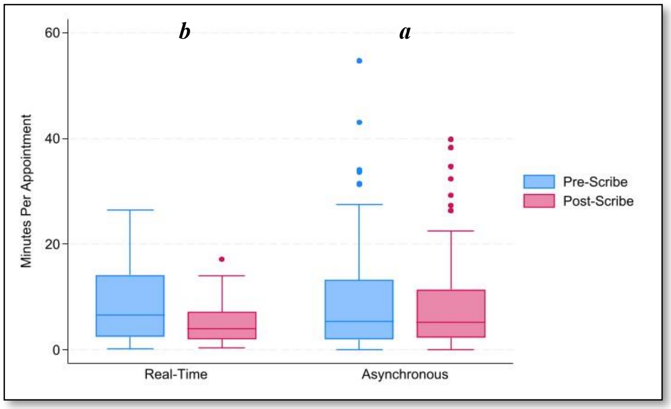

Proportion of Note by the Physician

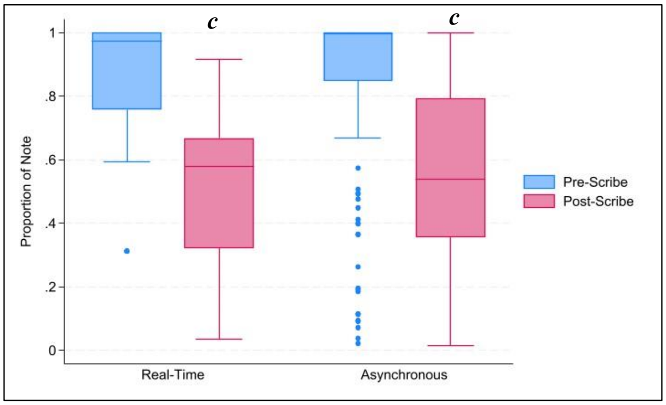

Proportion of Orders with Team Contribution

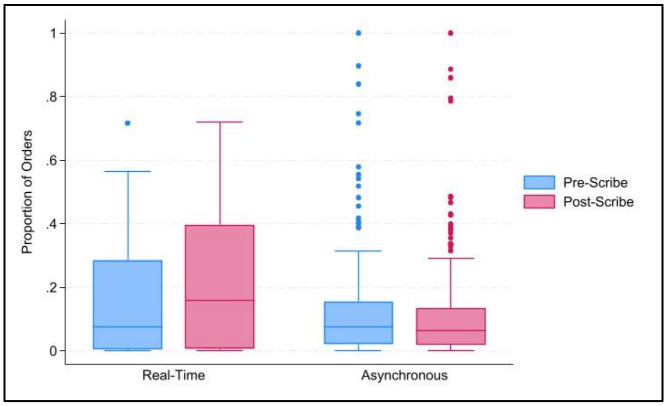

[a]p<0.05; [b]p<0.01; [c] p<0.001

© 2024 Rotenstein L et al. *JAMA Network Open.*

eFigure 3. Six-Month Change in EHR Metrics With Scribe Use for Overall Cohort

a) Total EHR Time Per Appointment

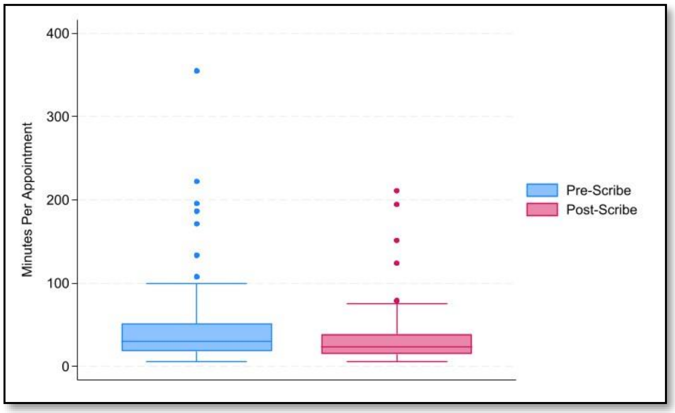

b) Note Time Per Appointment

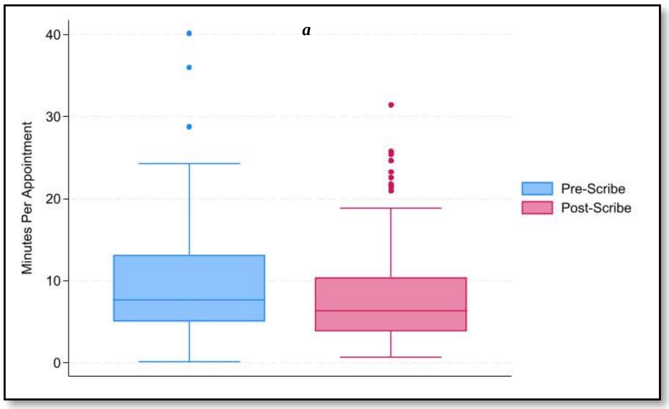

c) Pajama Time Per Appointment

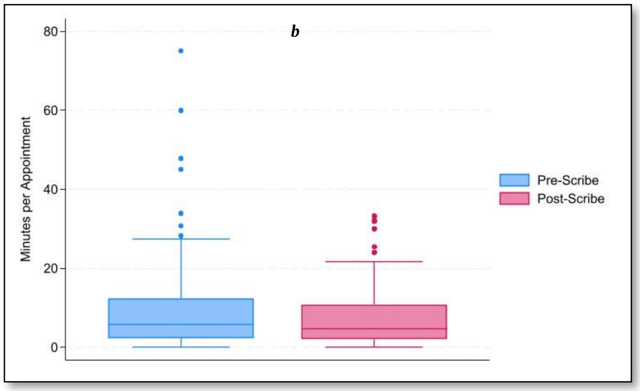

d) Percent of the Note by the Physician

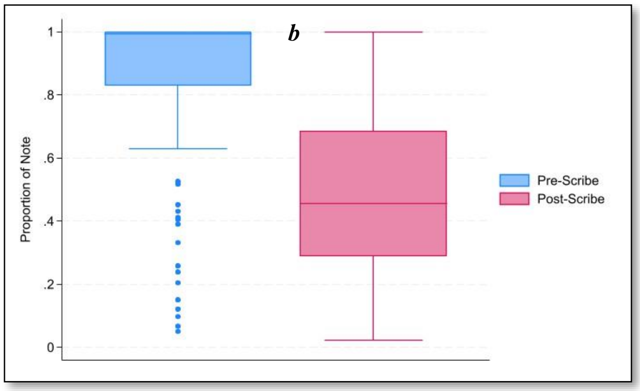

e) Proportion of Orders with Team Contribution

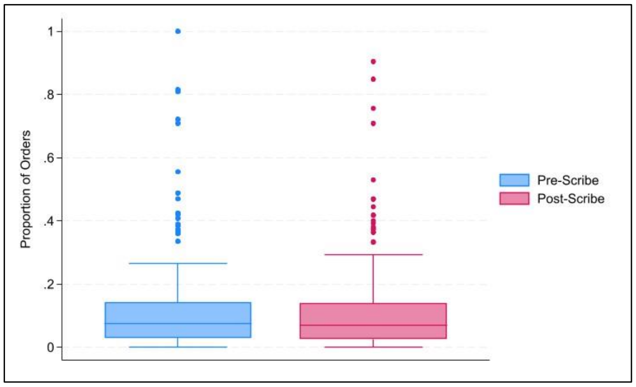

[a] p<0.001, [b] p<0.01

All p-values corrected for multiple comparisons using the Benjamini-Hochberg method

© 2024 Rotenstein L et al. *JAMA Network Open.*

eFigure 4. Six-Month Change in EHR Metrics With Scribe Use, Stratified by Specialty

a) Total EHR Time Per Appointment

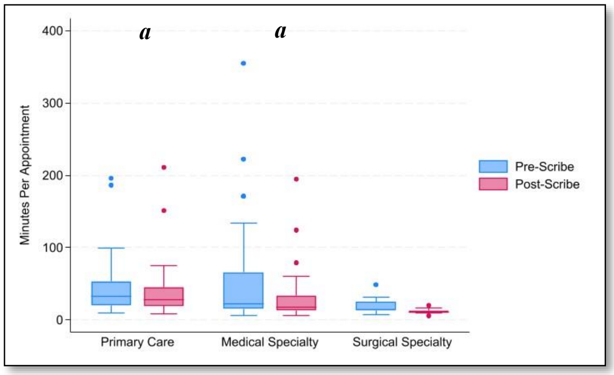

b) Note Time Per Appointment

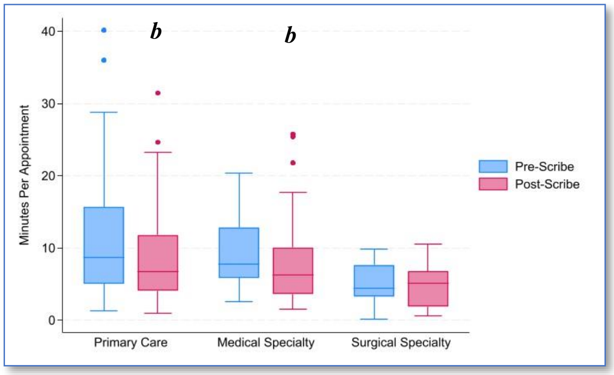

c) Pajama Time Per Appointment

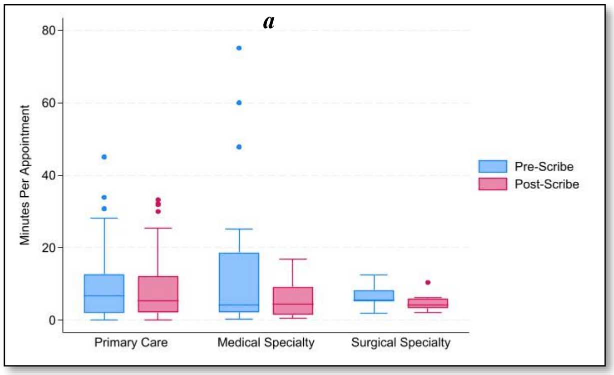

d) Percent of the Note by the Physician

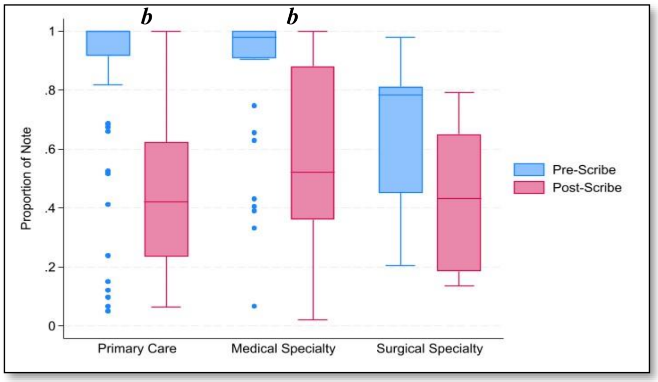

e) Proportion of Orders with Team Contribution

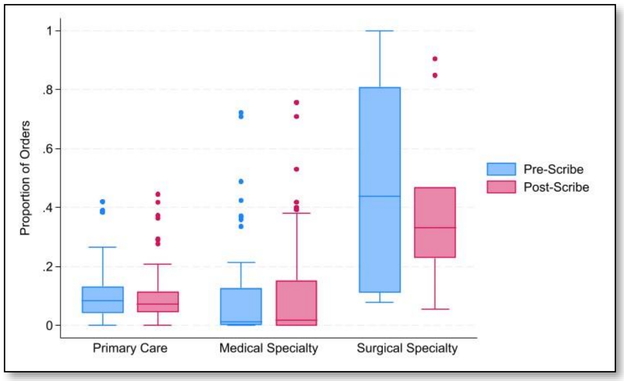

[a] p<0.05; [b] p<0.001
All p-values corrected for multiple comparisons using the Benjamini-Hochberg method

© 2024 Rotenstein L et al. *JAMA Network Open.*

eFigure 5. Six-Month Change in EHR Metrics With Scribe Use, Stratified by Scribe Service Type

a) Total EHR Time Per Appointment

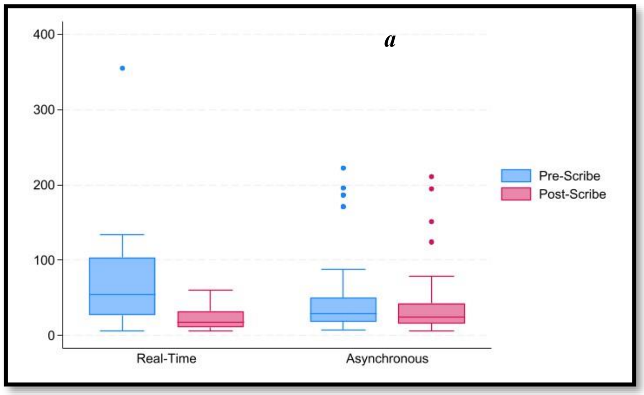

b) Note Time Per Appointment

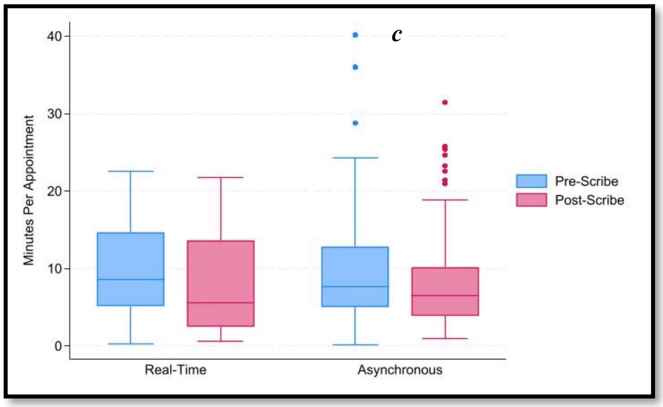

c) Pajama Time Per Appointment

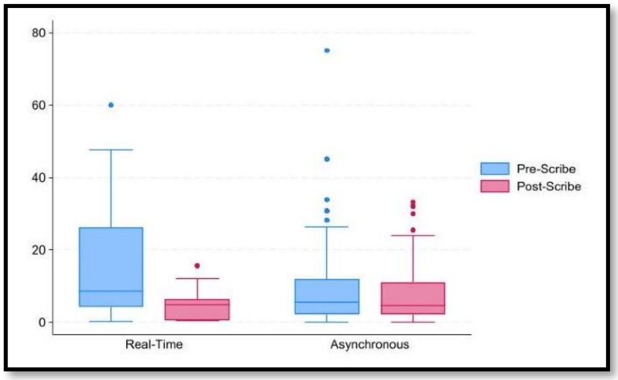

d) Percent of the Note by the Physician

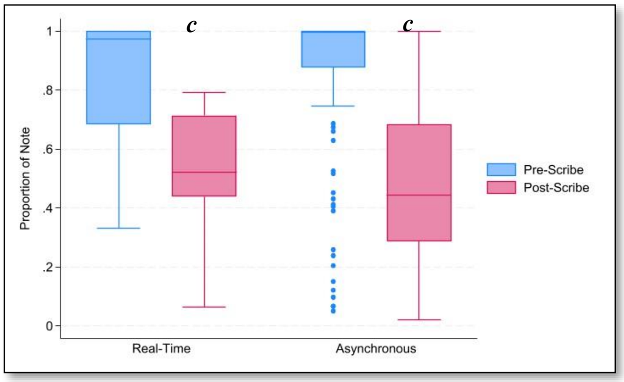

e) Proportion of Orders with Team Contribution

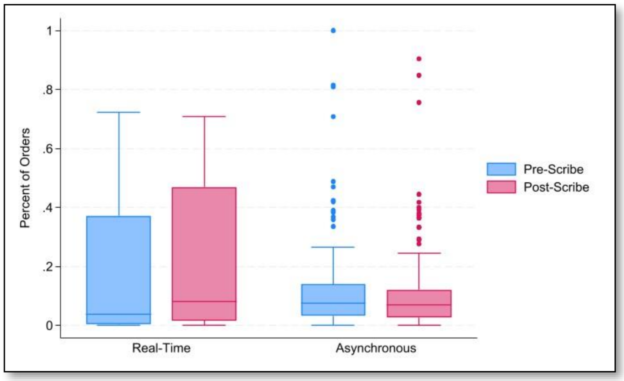

[a] p<0.05; [b] p<0.01; [c] p<0.001
All p-values corrected for multiple comparisons using the Benjamini-Hochberg method

© 2024 Rotenstein L et al. *JAMA Network Open.*

eTable 3. Factors Associated With 6-Month Change in a) Total EHR Time; b) Notes Time; c) Pajama Time, all Per Appointment, After Scribe Use

	Total EHR Time Per Appointment		Notes Time Per Appointment		Pajama Time Per Appointment	
**Parameter**	**Estimate (95% CI), Minutes Per Visit**	**P-Value**	**Estimate (95% CI), Minutes Per Visit**	**P-Value**	**Estimate (95% CI), Minutes Per Visit**	**P-Value**
Baseline EHR Time Per Appointment (Minutes)	-0.7 (-0.8, -0.6)	<0.0001	-0.3 (-0.4, -0.2)	<0.0001	-0.6 (-0.7, -0.5)	<0.0001
Change in Percent of Note Contribution by the Provider	8.5 (-5.0, 22.1)	0.22	4.9 (3.0, 6.8)	<0.0001	2.3 (-1.2, 5.9)	0.20
**Specialty**						
Medical Specialty	-17.9 (-27.9, -7.9)	0.0005	-1.2 (-2.6, 0.3)	0.11	-4.4 (-7.0. -1.8)	0.0001
Surgical Specialty	-17.8 (-34.1, -1.6)	0.03	-0.6 (-2.9, 1.7)	0.59	-1.9 (-6.0, 2.2)	0.36
Primary Care	-	-			-	-
**Scribe Service Type**						
Asynchronous	7.1 (-7.7, 21.8)	0.35	-1.2 (-3.2, 0.8)	0.26	2.3 (-1.4, 6.0)	0.22
Real-Time Scribe	-	-			-	-
**Institution**						
BWH	-0.9 (-13.4, 11.5)	0.88	0.1 (-1.6, 1.8)	0.92	1.4 (-1.7, 4.6)	0.38
MGH	-	-			-	-
**PCP Gender**						
Female PCP	1.5 (-7.1, 10.1)	0.74	-0.2 (-1.4, 1.1)	0.78	0.9 (-1.3, 3.2)	0.42
Male PCP	-	-			-	-
**Years Since Residency**						
0 to 10 years since residency	-2.1 (-11.4, 7.2)	0.66	-0.3 (-1.6, 1.0)	0.68	0.1 (-2.3, 2.5)	0.92
11 to 20 years since residency	-5.0 (-15.7, 5.7)	0.36	-0.1 (-1.6, 1.4)	0.88	0.4 (-2.4, 3.2)	0.77
> 20 years since residency	-	-			-	-
**Change in the Percent of Orders with Team Contribution**	-17.9 (-72.7, 26.8)	0.43	0.4 (-5.9, 6.7)	0.89	-1.0 (-12.4, 10.5)	0.87

© 2024 Rotenstein L et al. *JAMA Network Open.*

eTable 4. Factors Associated With 6-Month Change in Proportion of the Note by the Physician

Parameter	Estimate (95% CI), Minutes Per Visit	Pr > |t|
Baseline Percent of Note by the Physician	-0.74 (-0.94, -0.55)	<0.0001
Baseline Note Time Per Appointment	0.01 (0.00, 0.01)	0.13
**Specialty**		
Medical Specialty	0.04 (-0.08, 0.16)	0.48
Surgical Specialty	0.08 (-0.11, 0.27)	0.40
Primary Care	-	-
**Scribe Service Type**		
Asynchronous	0.01 (-0.16, 0.17)	0.13
Real-Time Scribe	-	-
**Institution**	0.01 (-0.13, 0.16)	0.87
BWH		
MGH	-	-
**PCP Gender**		
Female PCP	0.02 (-0.08, 0.13)	0.70
Male PCP	-	-
**Years Since Residency**		
0 to 10 years since residency	0.03 (-0.08, 0.14)	0.63
11 to 20 years since residency	0.01 (-0.12, 0.13)	0.90
> 20 years since residency	-	-

© 2024 Rotenstein L et al. *JAMA Network Open.*

eFigure 5. Pajama Time per Appointment by Scribe Type

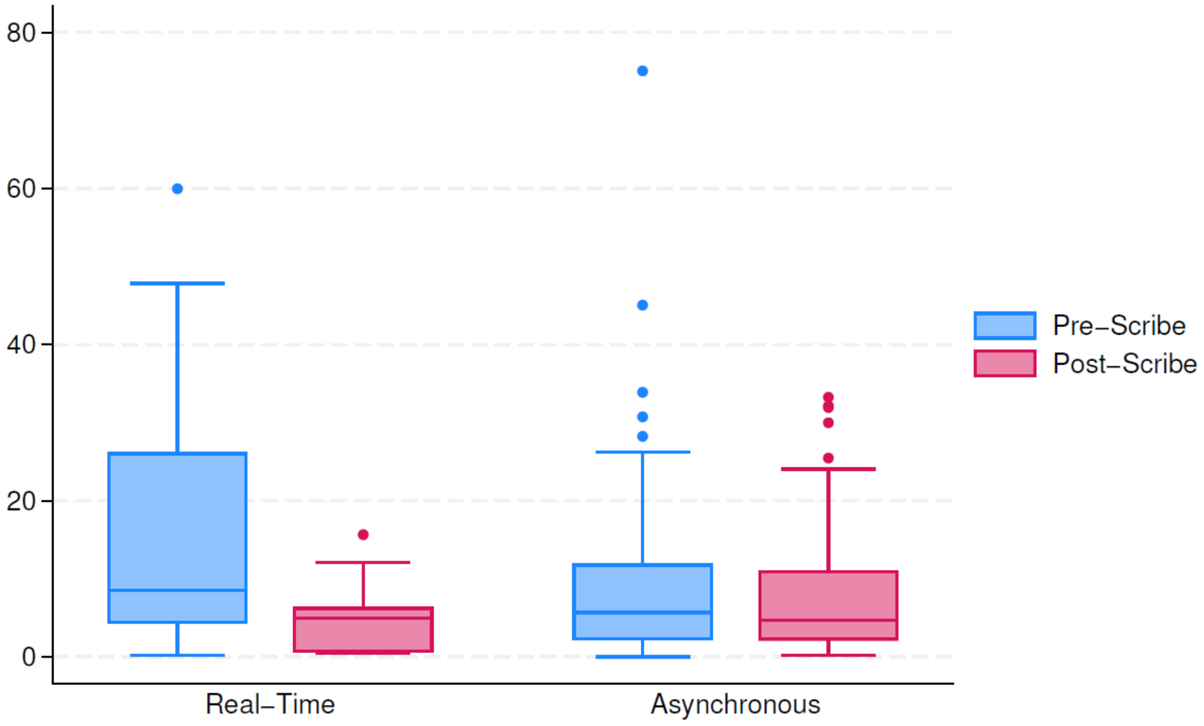

© 2024 Rotenstein L et al. *JAMA Network Open.*
