# Supplementary material for: Virtual Scribes and Physician Time Spent on Electronic Health Records
Source: JAMA Netw Open. 2024 May 24;7(5):e2413140. doi: 10.1001/jamanetworkopen.2024.13140 (PMC11127114; doi:10.1001/jamanetworkopen.2024.13140)
Supplement: Supplement 2. — Data Sharing Statement [file jamanetwopen-e2413140-s002.pdf]

Data Sharing Statement

Rotenstein. Virtual Scribes and Physician Time Spent on Electronic Health Records. *JAMA Netw Open*. Published May 24, 2024. doi:10.1001/jamanetworkopen.2024.13140

Data
Data available: No

Additional Information
Explanation for why data not available: This is proprietary health system data.
